# Thyroid dysfunction and cardiovascular events in patients with chronic kidney disease: A protocol of systematic review and meta-analysis

**DOI:** 10.1097/MD.0000000000023218

**Published:** 2020-11-20

**Authors:** Tongtong Liu, Yingjie Guan, Juan Li, Huimin Mao, Yongli Zhan

**Affiliations:** a Guang’anmen Hospital, China Academy of Chinese Medical Sciences; b Centre for Evidence-Based Chinese Medicine, Beijing University of Chinese Medicine, Beijing, China.

**Keywords:** cardiovascular events, chronic kidney disease, protocol, systematic review, thyroid dysfunction, trial sequential analysis

## Abstract

**Background:**

Cardiovascular disease is the main cause of death in patients with chronic kidney disease (CKD). Studies have found that hypothyroidism can significantly increase cardiovascular risk. Meanwhile, hypothyroidism is a common complication of CKD, but the correlation between hypothyroidism and cardiovascular risk in CKD patients has not been verified and paid enough attention. We therefore plan to conduct a systematic review and meta-analysis to explore whether hypothyroidism was independently predictive for the cardiovascular risk in patients with CKD.

**Methods:**

We will search in PubMed, Embase Database, Web of Science, China National Knowledge Infrastructure (CNKI), China Biology Medicine Database (CBM), and Wanfang Database, and include the cross-sectional studies, case--control studies, and cohort studies that explore the association between hypothyroidism and cardiovascular risk in CKD patients. According to the eligibility criteria, two researchers will independently screen the retrieved literature, evaluate the methodological quality, and extract data. We will combine the extracted data based on STATA and TSA software.

**Results:**

This systematic review will assess the association between hypothyroidism and cardiovascular risk in CKD patients based on the incidence of cardiovascular events in CKD people with hypothyroidism.

**Conclusions:**

This study will provide more evidence for the correlation between hypothyroidism and cardiovascular risk in CKD patients, which will contribute to the management and clinical practice of CKD population.

**Ethics and dissemination:**

This protocol is based on available literatures so that the ethical approval and informed consent are not applicable. The results of this study will be published in a peer-reviewed journals or relevant conferences.

**Protocol registration number:**

INPLASY2020100022.

## 1 Introduction

Chronic kidney disease (CKD) is an irreversible and progressive disease that emerging as a global epidemic. The incidence of CKD increases year by year. The estimated global prevalence of CKD is 11% to 13%.^[1]^ In 2017, about 697.5 million population suffered from CKD worldwide.^[2]^ Cardiovascular disease (CVD) is one of the leading causes of death in adults with CKD. Patients always suffer from CKD and CVD at the same time, It is widely believed that some common risk factors for CKD, such as hypertension, diabetes mellitus, hypertension and dyslipidemia are also important reasons for the progression of CVD. Recently, more and more potential risk factors related to the risk of CVD in CKD patients have been widely concerned, such as hypothyroidism.

Notably, hypothyroidism has been proved to be associated with the progression of many diseases, such as, nonalcoholic fatty liver disease (NAFLD),^[3]^ fracture,^[4]^ and systemic lupus erythematosus,^[5]^ etc. CVDs^[6]^ are no exception. Indeed, studies have shown that hypothyroidism can lead to dyslipidemia, endothelial dysfunction, and blood pressure changes, etc^[7–9]^, which result in to the increased long-term vascular risk. Hypothyroidism is a common complication in CKD, and the risk of renal function loss in CKD patients with hypothyroidism has significantly increased.^[10,11]^ More importantly, Huang et al^[12]^ conducted a follow-up study on 2103 participants without CKD and found that hypothyroidism is associated with an increased risk of incident CKD. However, the correlation between hypothyroidism and cardiovascular risk in CKD patients has not been recognized and paid enough attention. We therefore plan to conduct a systematic review and meta-analysis, based on the available studies, to address the association between hypothyroidism and cardiovascular events (CVEs) in CKD patients. Our objective was to assess whether hypothyroidism was independently predictive for the cardiovascular risk in patients with CKD.

## 2 Methods

The protocol of systematic review and meta-analysis has been registered on the International Platform of Registered Systematic Review and Meta-analysis Protocols

(INPLASY) as INPLASY2020100022. The preferred reporting items for systematic review and meta-analysis protocols (PRISMA) checklist will be used as a guideline for the implementation of this study and subsequent reporting.^[13]^

### 2.1 Eligibility criteria

#### 2.1.1 Types of studies

All cross-sectional, case—control, or cohort studies that explore the association of hypothyroidism and the risk of CVD in patients with CKD will be included in this systematic review, and repetitive literature, meta-analysis, case reports, reviews, conference abstracts, comments, protocols, and practice guidelines will be excluded. There are no restrictions on language and publication.

#### 2.1.2 Types of participants

All patients included in this study must be diagnosed with CKD. The diagnostic criteria for CKD is estimated glomerular filtration rate (eGFR) <60 mL/min/1.73 m^2^, or with albuminuria,^[14]^ without restrictions on country, gender, race, age, or course of disease. We will exclude the study that participants had acute or chronic diseases other than CKD in this study.

#### 2.1.3 Types of interventions

The research we need is to compare patients with hypothyroidism and those without hypothyroidism in the CKD population. The diagnostic criteria of hypothyroidism are based on clinical symptoms, signs, and laboratory indicators. Clinical hypothyroidism is defined as increased serum thyroid-stimulating hormone (TSH) levels with decreased free thyroxine (FT4) levels, and subclinical hypothyroidism is defined as elevated TSH levels with normal FT4.^[15]^ It is important that we will exclude this study if hypothyroidism is not the main influencing factor in this study.

#### 2.1.4 Types of primary outcome

The primary outcome is CVE, including acute myocardial infarction, angina pectoris, readmission for myocardial infarction or heart failure, need to undergo coronary artery bypass surgery, and cardiovascular death, etc,^[16]^ and each included study must contain at least one outcome indicator. Besides, study that cannot extract valid data will be excluded.

### 2.2 Data sources and searches

**Figure 1:**
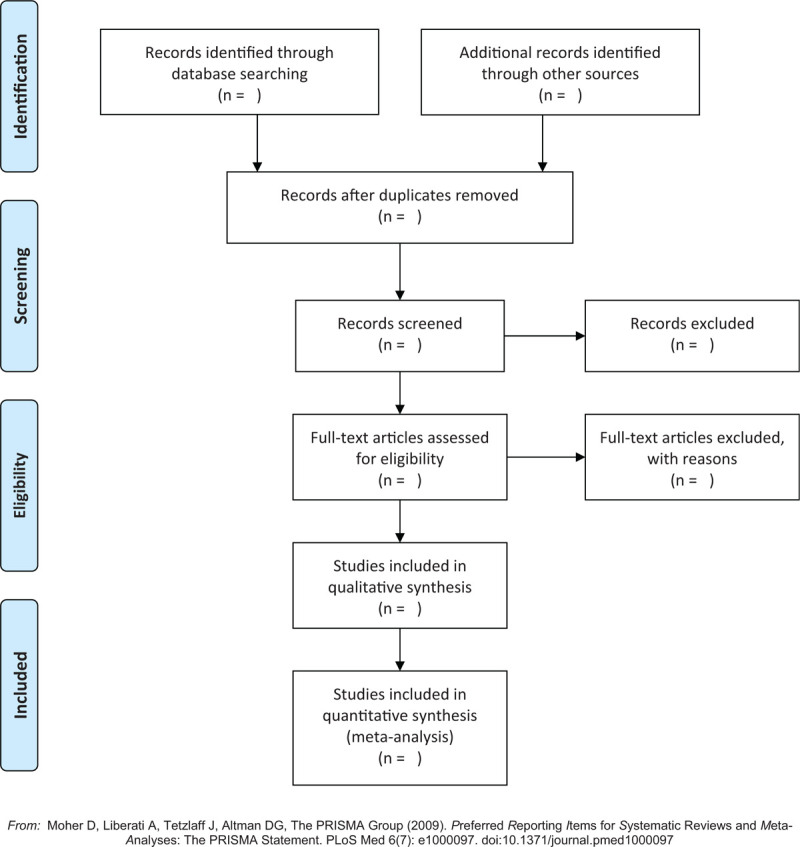
The flow diagram of literature screening process.

On the basis of PubMed, Embase Database, Web of Science, China National Knowledge Infrastructure (CNKI), China Biology Medicine Database (CBM), and Wanfang Database, we conducted a systematic search of the literature on the association of hypothyroidism and the risk of CVD in patients with CKD. The retrieval strategy is the combination of Medical Subject Headings (MeSH) terms and free terms. In addition, we will complement studies from references of the retrieved literature to make the search more comprehensive. The retrieval formula of PubMed is summarized in Table 1, and the literature screening process is shown in Figure 1.

**Table 1:** Retrieval formula of PubMed.

Number	Search items
#1	(((((((((Chronic Kidney Disease[MeSH Terms]) OR (Chronic Renal Disease[Title/Abstract])) OR (Kidney Disease, Chronic[Title/Abstract])) OR (Renal Disease, Chronic[Title/Abstract])) OR (Disease, Chronic Kidney[Title/Abstract])) OR (Disease, Chronic Renal[Title/Abstract])) OR (Kidney Insufficiency, Chronic[Title/Abstract])) OR (Renal Insufficiencies, Chronic[Title/Abstract])) OR (Chronic Kidney Insufficiency[Title/Abstract])) OR (Chronic Renal Insufficiency[Title/Abstract])
#2	((((Hypothyroidism[MeSH Terms]) OR (Thyroid Dysfunction[Title/Abstract])) OR (Thyroid hormones[Title/Abstract])) OR (Thyroid stimulating hormone[Title/Abstract])) OR (free thyroxine[Title/Abstract])
#3	(((cardiovascular events[Title/Abstract])) OR (cardiovascular mortality[Title/Abstract])) OR (MACE[Title/Abstract])
#4	#1 AND #2 AND #3

### 2.3 Data extraction

The information extraction of included literatures will be carried out independently by 2 researchers (YJ GUAN and J LI). The contents of literature extraction include basic information: first author, author's unit (in order to avoid the superposition effect of multiple studies in the same unit and the same patient population), year of publication, country, etc; characteristics of participants: diagnosis method, sample size, average age, sex ratio, average disease course, definition of hypothyroidism, etc; outcome indicators: types of outcome indicators, measurement methods, values, etc; quality assessment: selection, comparability, exposure, etc, if there are multiple studies for the same group of patients, we only include the latest one with the largest sample size.

### 2.4 Quality assessment

Two investigators (TT LIU and HM MAO) independently evaluated the quality of the included literature according to the Newcastle-Ottawa Scale (NOS)^[17]^ for case--control studies and cohort studies and Agency For Healthcare Research and Quality (AHRQ)^[18]^ for cross-sectional studies. If there is divergence in the evaluation between the 2 researchers, the decision will be made through consultation with the third researcher (YL ZHAN). The main contents of the assessment are as follows: the accuracy of diagnosis in selected cases, the representativeness of selected cases, comparability between the observation group and the control group, the quality of factor exposure in case--control studies, and follow-up adequacy of outcome indicators in cohort studies.

### 2.5 Grading the quality of evidence

Two researchers (TT LIU and HM MAO) will independently assess the quality of evidence for each outcome indicator via the Grading of Recommendations Assessment, Development, and Evaluation (GRADE). ^[19]^ If there is inconsistency in the evaluation between the 2 researchers, the decision will be made through consultation with the third researcher (YL ZHAN). All outcome indicators will be graded into 4 levels based on the quality of evidence: high, moderate, low, and very low quality. The reasons for the degradation of evidence quality of all outcome indicators will be commented.

### 2.6 Data synthesis and analysis

The risk ratio (RR) and 95% confidence intervals (95% CIs) will be used as the effect values of binary variables, and the mean difference (MD) and 95% CI will be used as the effect values of continuous variables. The random effect model will be used to calculate the effect size, as this method can better integrate the potential differences between studies, whether there is significant heterogeneity or not. Chi-squared test and *I*^2^ will be used to evaluate the heterogeneity among the studies (low heterogeneity is defined as *P* > .05, *I*^2^ < 50%, medium heterogeneity is defined as *P* close to .05 and *I*^2^ close to 50%, high heterogeneity is defined as *P* < .05, *I*^2^ > 50%^[20]^).

We will use the software of STATA (version 16.0) for meta-analysis, subgroup analysis, sensitivity analysis, and publication bias test. Trial sequential analysis (TSA, version 0.9.5.10) will be used to conduct TSA of this study. The significance of all statistical tests will be set as *P* < .05.

### 2.7 Subgroup analysis

If there is a high heterogeneity between studies, subgroup analysis will be conducted to identify possible sources of heterogeneity, such as, age, gender, race, disease stage, type of study, quality of literature, etc.

### 2.8 Sensitivity analysis

We will eliminate the low-quality literature one by one to observe the stability of the research results to complete the sensitivity analysis of this study.

### 2.9 Publication bias

We will evaluate the possible publication bias qualitatively and quantitatively by additional contour funnel plot and Egger test.

### 2.10 Trial sequential analysis

TSA will be used to evaluate the reliability and stability of the conclusions.^[21]^ We will set type I error as 5%, and the power as 80% for the calculation of the required information size (RIS). If the cumulative *Z* curve crossed the traditional boundary value and RIS, it means that the results obtained by this meta-analysis have strong stability and credibility.

### 2.11 Ethics and dissemination

There is no need for ethical support or informed consent for this protocol of systematic review, because this research is based on published literature. Our final results will be published in peer-reviewed journals and disseminated through relevant conferences.

## 3 Discussion

The development and progression of CVD are very closely related to CKD. The incidence of CVD in CKD patients is higher than that in the general population, even in the early stage of CKD, and the cardiovascular risk increase significantly with the decline of renal function. CKD patients with CVEs have worse prognosis and higher mortality.^[22]^ Hypothyroidism is highly prevalent in patients with CKD, and it may coexist with CKD, mutual influence, aggravate each other, and jointly increase the cardiovascular risk. Rhee et al^[23]^ followed up 2715 dialysis patients and found a significant correlation between hypothyroidism and all-cause mortality. In CKD patients, hypothyroidism may not only cause hemodynamic changes, but also cause vascular calcification and endothelial damage, which are the pathologic basis of CVD^[24]^; this suggests that hypothyroidism may be associated with severe cardiorenal damage. However, hypothyroidism has not been paid enough attention in CKD management and clinical practice. Therefore, this study tends to explore the correlation between hypothyroidism and cardiovascular risk in patients with CKD, in order to provide better evidence for CKD management and clinical practice.

## Author contributions

**Conceptualization:** MM Huimin Mao, Tongtong Liu.

**Data curation:** MM Yingjie Guan, MM Juan Li.

**Formal analysis:** Tongtong LIU, MM Yingjie Guan.

**Investigation:** MM Juan Li, Yingjie Guan

**Methodology:** Tongtong Liu, MM Yingjie Guan.

**Supervision:** MM Huimin Mao, Yongli Zhan.

**Validation:** Tongtong Liu, MM Yingjie Guan, MM Huimin Mao.

**Writing – original draft:** Tongtong Liu, MM Yingjie Guan.

**Writing – review & editing:** MM Huimin Mao, Yongli Zhan.
